# Human–Co-Bot Interaction and Neuroergonomics: Co-Botic vs. Robotic Systems

**DOI:** 10.3389/frobt.2021.659319

**Published:** 2021-05-04

**Authors:** Federico Cassioli, Giulia Fronda, Michela Balconi

**Affiliations:** ^1^International Research Center for Cognitive Applied Neuroscience (IrcCAN), Catholic University of the Sacred Heart, Milan, Italy; ^2^Research Unit in Affective and Social Neuroscience, Department of Psychology, Catholic University of the Sacred Heart, Milan, Italy

**Keywords:** co-bot, industry 4.0., human-robot interaction, neuroergonomics, applied neuroscience

## Human–Robot Interaction: A Technical and Managerial Matter

The fourth industrial revolution comprehends smart manufacturing, where sensors, computing platform, and data modeling are employed (Kusiak, 2018). Di Nardo et al. (2020), in the frame of Industry 4.0, developed a model where the role of management is key in this new highly networked environment. It is suggested that cyber-physical systems, along with massive data acquisition and mining, might support the decision making and planning execution phases.

In this framework, technological advancements are a necessary, but not sufficient condition. In fact, a functional and targeted human–machine interaction, defined as a communication/interaction between the human user and machines via different interface in a dynamic context, is also essential.

Management has to oversee the rising demand for tech-innovation, which is essential because of the renewed complexity, the stricter time-to-market process, and a higher competition generated by globalization (De Carolis et al., 2016), and to ensure that innovation fits well within the work environment. In this sense, the automation of part of the process adds value only if substantial changes are implemented among all the organization, which happens when the efficiency of the machine is strengthened by human cognitive skills and adequate flexibility. Under this light, neuromanagement, a new branch of management, was recently developed, where decision-making processes (Balconi and Fronda, 2019, 2020a) and social behavior and interaction (Balconi and Vanutelli, 2017; Venturella et al., 2017; Balconi and Fronda, 2020b) are studied in real-world situations by using a neuroscientific approach.

The conjunction and the outcome of this multidisciplinary approach might boost smart manufacturing, in particular for co-bot technology, where operational fluency between agents has a significant weight for safety and productivity reasons. In this work, with the term “co-bot,” we intend to underline its collaborative dimension, being it the main feature that differentiates from other technological systems (Ajoudani et al., 2018).

## Co-Bots for the Industry: Role and Applications

Co-bots can be defined as novel technological manufacturing systems, which are able to work with a certain degree of dexterity and in conjunction with humans in the same physical workspace (Bauer et al., 2016), with no barriers, mainly aiming at improving efficiency, flexibility, and quality in the overall industrial process. Other possible appreciable dimensions are related to ergonomics and safety (Kildal et al., 2018), being the co-bot mostly responsible and employable for monotonous and unergonomic tasks. Regarding the safety, it must be pointed out that Industry 4.0 brings also emerging risks and challenges, which are related to the human performance (Brocal et al., 2019).

More generally, according to the International Federation of Robotics (IFR), co-bot technology might help in two different contexts. In the small- to medium-sized companies, it could be introduced to automate some parts of the production line, without altering the rest and offering higher productivity and quality improvements. Second, in companies with already automated process (e.g., automotive sector), it could support workers in completing assemblage tasks, often causing physical injuries. Regarding the market data, in 2019, the professional service robots sector grew by 32% (from US $8.5 billion to $11.2 billion) (Executive Summary World Robotics, 2020), and the sales volume for collaborative robots grew more compared to the traditional ones (IFR Press Conference, 2020). Also, the pandemic seems to have boosted the market for robotic components in warehouses, factories, and home delivery and also because the technology supports social distancing.

However, some important differences should be considered and elucidated when comparing “robotic” and “co-botic” systems, focusing on the level of interaction with the workers, higher for the co-botic compared to the robotic one, which is physically separated and has a fixated position.

The recent developments in sensors and data processing led to systems that better assist and interact with humans (e.g., Fryman and Matthias, 2012). Although, fully collaborative co-bot applications are not completely developed and used yet, and there is a significantly high variance in the technical applications of co-bot. In fact, depicting a hypothetical continuum for human–robot collaboration, from no direct human–robot contact to a real-time system that adjusts in response to the human behavior, it is most common to have just shared workspace and/or sequential collaboration conditions.

A successful industrial adoption of co-bots is derived from proper training programs and an open communication that address the company objectives. In the literature, three categories of factors are highlighted: internal (management support, company structure, research, physical conditions, and receptiveness), external (regulatory environment, business partner), and technological (technology context, degree of innovation, and workspace) (Correia Simões et al., 2020). Besides the delicate coexistence of operational efficiency and safety requirements, another possible issue, as suggested by Bauer et al. (2016), because of the novelty of co-bot technology, is that old models assessing efficiency and profitability fail to give a proper cost–benefit analysis, and these are not usually carried out by companies. Furthermore, some dimensions, such as ergonomics, stress, flexibility, and relationship data, are difficult to be measured and quantified.

## Discussion: Neuroergonomics in the Organization

The new paradigm advocates for an optimized human–robot interaction (HRI), where robots carry out a fully collaborative behavior, and both the strengths of the involved agents are maximized. As already mentioned, previous approaches to HRI showed difficulties in the measurement and quantification of some dimensions such as ergonomics and interaction. Other previous theoretical frameworks were proposed (e.g., Goodrich and Schultz, 2007). In particular, a more novel approach (Gervasi et al., 2020) postulates that collaborative systems can be evaluated by combining both technical aspects with human social factors and highlights eight latent dimensions, such as information exchange, autonomy, adaptivity and training, human factors, ethics, team organization, task, and cyber-security. In light of these contributions, we believe that, with such set goal, the adoption of neuroergonomics, for its study of neural networks involving cognitive, perceptual, and emotional processing and, in general, applied neuroscience, is mandatory to be considered.

As a result of the increased and renewed portability of brain–computer interface (BCI), at reasonable cost, neurophysiological and behavioral sensors can be useful for the development of fully collaborative co-bots into the industrial context. In fact, some of the weighting factors that should be considered in the developed of co-bots are human fatigue, as a function of time and workload, and executive functions (in particular working memory, inhibition, and cognitive flexibility), which are responsible for dynamic attentional coordination and are impaired by stress (e.g., Shields et al., 2016), selective attention, and cognitive states.

Indeed, each of these factors may better explain the usefulness, applicability, and quantitative impact of co-botic systems in real workplaces. Specifically, regarding fatigue, the optimization process and the management of adjusting robots' trajectories could facilitate the human operator's work. In this regard, to reduce worker's fatigue, elements such as the condition of stability, the possible constraints of the activities, and the presence of shared workspaces should be considered (Kim et al., 2018; Hashemi-Petroodi et al., 2020). The presence of co-bots in the industrial context could also be effective in terms of performance, allowing better use of resource skills and executive functions (Tsarouchi et al., 2016). Indeed, the advantages introduced by the inclusion of robots with characteristics such as strength, speed, precision, tirelessness, and repeatability will allow reduced cognitive load and effort for the workers performing their duties and allowing better use of intelligence, creativity, and learning (Hashemi-Petroodi et al., 2020).

To assess mentioned dimensions in the co-bot, we highlighted and propose some of the neurometrics that respond to the purpose. A major distinction that should be taken into consideration is the parameter domain, meaning if it refers to the central electroencephalography (EEG) and event-related potentials (ERPs), peripheral [electrodermal activity (EDA), electrocardiogram data, respiratory system], or behavioral (mostly derived from visual eye and gaze tracking systems) system. The consideration of these parameters, consisting in the detection and processing of sensory data, could allow co-bots to more easily understand the objectives and intentions of human partners and assist them in carrying out specific tasks.

Regarding the mental load, many studies used slow-wave and fast-wave increases/decreases and (α/θ)/β or (α/θ)/(α + β) ratios in the frontal and central brain areas (e.g., Wang et al., 2020) to explore the brain networks contribution in cognitive and emotional planning. In parallel, information about emotion recognition has been collected via frontal asymmetry (Balconi and Mazza, 2010; Balconi et al., 2014), normalized frontal asymmetry (Balconi et al., 2009, 2015), theta-beta ratio (Angelidis et al., 2018), and Hjorth parameters for affective state estimation (e.g., Rakibul Mowla et al., 2020).

Regarding selective attention, instead, ERP approach can be widely used to study the degree of attention, employing P1 and N1 and later components such as P300, reflecting, among the others, the identification of a target. In addition to cerebral outputs, also behavioral measures can be collected, as the identification of stimulus-driven saccades, time-to-first fixations, and pupil dimension, which might be very informative about visual attentional behavior and the overall representation of the workers of their body position, movement, and acceleration in the workplace.

On the other side, always more novel and varied techniques are applied to biosignal and behavioral research (Cassioli and Balconi, 2020), aiming at classifying, reducing the dimensionality (Zhang et al., 2019), and predicting workers' behavior. The most notable is that the recent application of machine learning to neurophysiological signal seems encouraging. Some of the methods are artificial neural networks (Baldwin and Penaranda, 2012), k-nearest neighbors, support vector machine (Son et al., 2013), and decision trees techniques (Solovey et al., 2014; Wang et al., 2020), via deep learning brain decoding techniques, showing that EEG signal might be used not only to obtain data but also as a support in the designing process through the use of brain activities, although it is important to note that actual data and models refer to limited dataset and set of categories. Also, intergroup differences might heavily limit the applications of these approaches.

**Figure 1:**
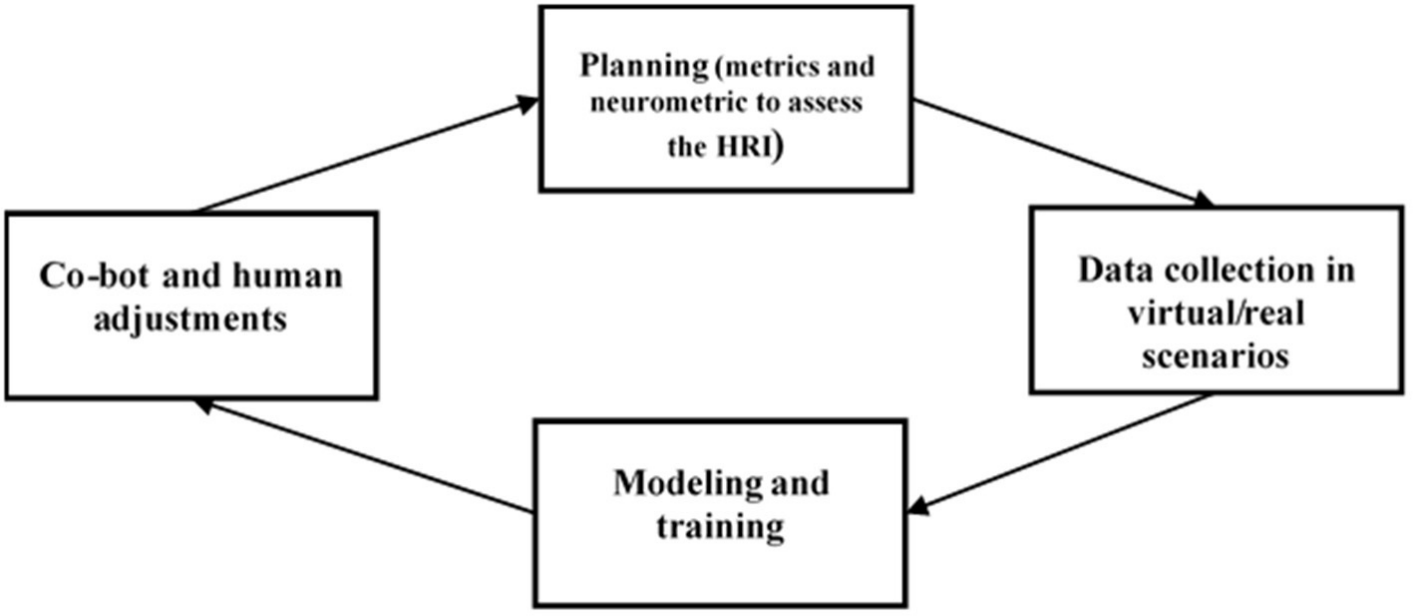
A hypothetical model applied to HRI for the industrial adoption of co-bots, based on the Deming cycle.

We then propose a quality cycle, structurally based, for the most part, on Deming cycle (Deming, 1986) and in line with the concept of neuroindustrial engineering coined by Ma et al. (2012) adjusted to the conjectured context for the development of an optimal HRI drawn in this work (Figure 1).

In the first phase (planning), a screening and a compartmentalization of the required processes are carried out. In this phase, it is important to establish objectives and the consequent BCI specifics (methods, chosen metrics) based on the procedural flow and set goals. In the second phase (doing), selected virtual and real scenarios are executed while data are collected. As in co-bot systems the fluidity of interaction and safety are primary requirements, we advocate for the application of a holistic approach and the joint consideration of central, peripheral, and behavioral parameters for the HRI evaluation. The following dimensions, among others, should be considered: the worker emotional discomfort, the executive functions (with a focus on irrelevant stimuli inhibition), the fatigue, and cognitive and emotional states in order to assist in decision-making processes. Also, an easy-to-use interface on which a feedback system is inserted should be provided in order to make the workers aware of their performance and status. Collected data is then (modeling/learning) used to create bottom-up models, which will be tested again in the next quality cycle. Finally, in the fourth phase, adjustments (change) are implemented in the workspace for both workers and the co-bots systems.

Furthermore, hyperscanning paradigms are now able to obtain data on actions and social adaptation during human-to-human interaction (Balconi et al., 2019a,b; Balconi and Fronda, 2020b). If portability will be increased, co-bots could receive precious information about multiple and complex work–environment settings with multiple agents.

Collaborative technology is still in its embryonic stage. We expect that further technological, neuroscientific, and behavioral developments will enrich and make this technology more intuitive, intelligent, and suitable for the human, leading to an optimized and safe human–co-robot interaction.

Because of the augmented portability of sensors and neuropshysiological systems, we believe that in the future smart manufacturing could adopt neuroscientific protocols to support workers on the field, aiming at increasing efficiency, ergonomics, and safety. In this work, we proposed a neuroindustrial quality process for the development of an optimized HRI for co-bot technology, based on the Deming cycle. We expect that further technological and neuroscientific developments will enrich and make co-bots more intuitive and suitable for the human.

## Author Contributions

FC, GF, and MB wrote the first draft and each section of the manuscript and contributed to the manuscript final writing and revision, read, and approved the submitted version. All authors contributed to the article and approved the submitted version.

## Conflict of Interest

The authors declare that the research was conducted in the absence of any commercial or financial relationships that could be construed as a potential conflict of interest.
